# Coproduction and enhancement of electricity and biobutanol using adsorption carrier solid-state fermentation

**DOI:** 10.1186/s13068-022-02138-6

**Published:** 2022-05-02

**Authors:** Xinyu Feng, Lan Wang, Hongzhang Chen

**Affiliations:** 1 State Key Laboratory of Biochemical Engineering, Beijing Key Laboratory of Biomass Refining Engineering, Institute of Process Engineering, Chinese Academy of Sciences, Beijing, 100190 People’s Republic of China; 2 University of Chinese Academy of Sciences, Beijing, 100049 People’s Republic of China

**Keywords:** Microbial fuel cell, Biobutanol, Adsorption carrier solid-state fermentation, Metabolic flux analysis, Extracellular electron transport

## Abstract

**Background:**

Electric energy is not collected and utilized in biobutanol fermentation. The reason is that the yields of electron shuttles and nanowires are not enough to gather and transfer all electrons to the electrode in liquid fermentation. However, the solid matrix of the adsorption carrier may be conducive to the collection and transfer of electrons because of its good adsorption and conductivity. Therefore, this first-attempt study coupled microbial fuel cell (MFC) with adsorption carrier solid-state fermentation (ACSF). In addition, the effect and mechanism of adsorption carrier solid-state fermentation on power generation were explored.

**Results:**

The power generation performance and fermentation performance were improved by ACSF. The power density by polyurethane and carbon felt carrier solid-state fermentation (PC) was 12 times that by no carrier fermentation (NC). The biobutanol yield of absorbent cotton and carbon felt carrier solid-state fermentation (ACC) was increased by 36.86%. Moreover, the mechanism was explored via metabolic flux analysis, cyclic voltammetry and scanning electron microscopy. The results of metabolic flux analysis showed that more electrons were produced and more carbon flowed to biobutanol production. The cyclic voltammetry results revealed that more riboflavin was produced to enhance extracellular electron transport (EET) by ACSF. The scanning electron microscopy image showed that the adsorption capacity and aggregation degree of bacteria were increased on the electrode and nanowires were observed by ACSF.

**Conclusions:**

A new fermentation mode was established by coupling MFC with ACSF to improve substrate utilization, which will provide crucial insights into the fermentation industry. In addition, the ACSF is an effective method to enhance power generation performance and fermentation performance.

**Graphical Abstract:**

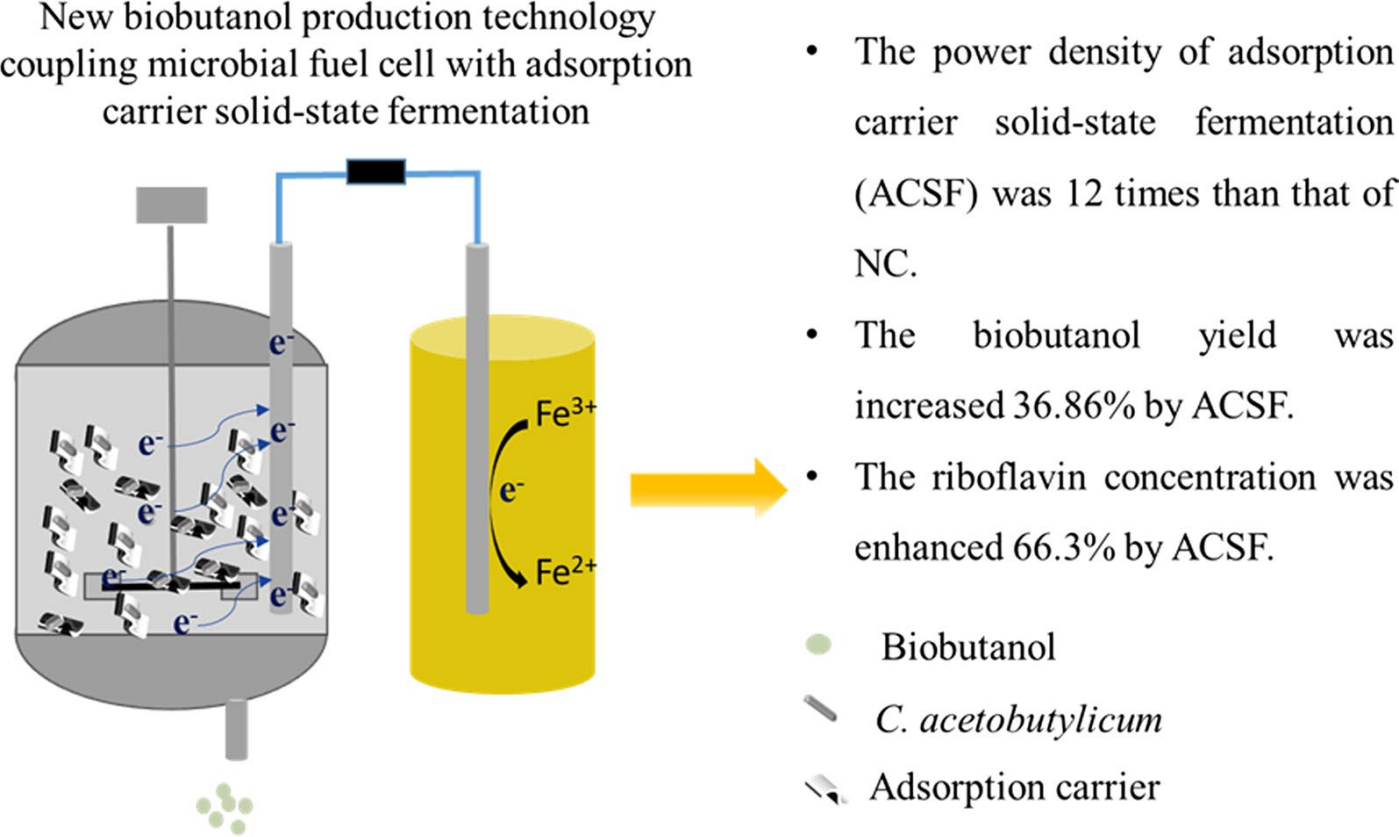

## Background

Biobutanol is considered to be a potential renewable fuel due to its high energy density, low volatility, low corrosiveness and environmental friendliness [1]. Therefore, the proposal and improvement of new biobutanol production technology has attracted the attention of many researchers. The coculture method of aerobic bacteria and anaerobic bacteria was proposed to improve butanol production and reduce energy consumption for maintaining an anaerobic environment [2]. In addition, carrier fermentation was also proposed to increase cell density and reduce substrate inhibition [3, 4]. Previous studies have shown that the cell concentration on activated carbon is 8 times that in suspend [5]. *Clostridium acetobutylicum* (*C. acetobutyricum*) is the main strain of producing butanol and also an electroactive microbe [6, 7].

*Clostridium acetobutyricum* was found to transfer electrons in the presence of electron shuttles in 1988 [8]. Then, *C. acetobutyricum* can transfer electrons to the electrode by direct electron transfer [9, 10]. This interesting finding means that not only high value-added chemicals, but also electricity in the fermentation process. This will change the previous understanding of simplified product types and bring new opportunities to biobutanol production technology development. However, how to collect and utilize electricity in the biobutanol fermentation process is a problem.

MFC is considered to be a potential strategy to improve substrate utilization. MFC is a new, green and low-cost power generation mode, which can convert chemical energy into electrical energy [11]. MFC will be an important part of renewable energy power generation power generation in the future. The produced electrons can be collected and utilized by applying MFC technology in the biobutanol production process. This new fermentation mode realizes the coproduction of electricity and biobutanol and changes the traditional biobutanol fermentation mode. In addition, it also broadens the MFC application range and accelerates industrialization. However, the disadvantages of liquid fermentation are more obvious after coupling MFCs with fermentation. These substances synthesized by electron and electron receptor junctions are disorderly dispersed and move slowly in the liquid matrix. This makes it difficult to gather electrons to the electrode, and then affects the output power density. This is also a factor that causes the power density of MFC to be 3 orders of magnitude lower than that of other types of fuel cells [12]. In addition, the liquid matrix easily leaks and has poor safety performance [13]. These factors greatly limit the application of MFC coupling biobutanol fermentation.

ACSF may be a promising method to couple MFC and biobutanol production. ACSF refers to the use of inert carrier materials as the solid phase in solid-state fermentation, which is similar to the biofilm reactor filled with packing/floating carriers. Solid-state fermentation is closer to the natural environment of bacterial growth, which is conducive to the growth of bacteria [14]. In addition, many solid matrix materials have good conductivity and adsorption. However, to our knowledge, few studies have attempted to explore the effect and mechanisms of ACSF on power generation performance.

In this work, biobutanol production and MFC power generation were coupled together in ACSF for the first time. The electrical performance and fermentation performance of MFCs were evaluated in ACSF. In addition, the mechanism of ACSF was analysed by metabolic engineering, cyclic voltammetry curves (CV) and scanning electron microscopy (SEM).

## Results and discussion

### Electrical performance of MFCs in adsorption carrier solid-state fermentation

**Fig. 1:**
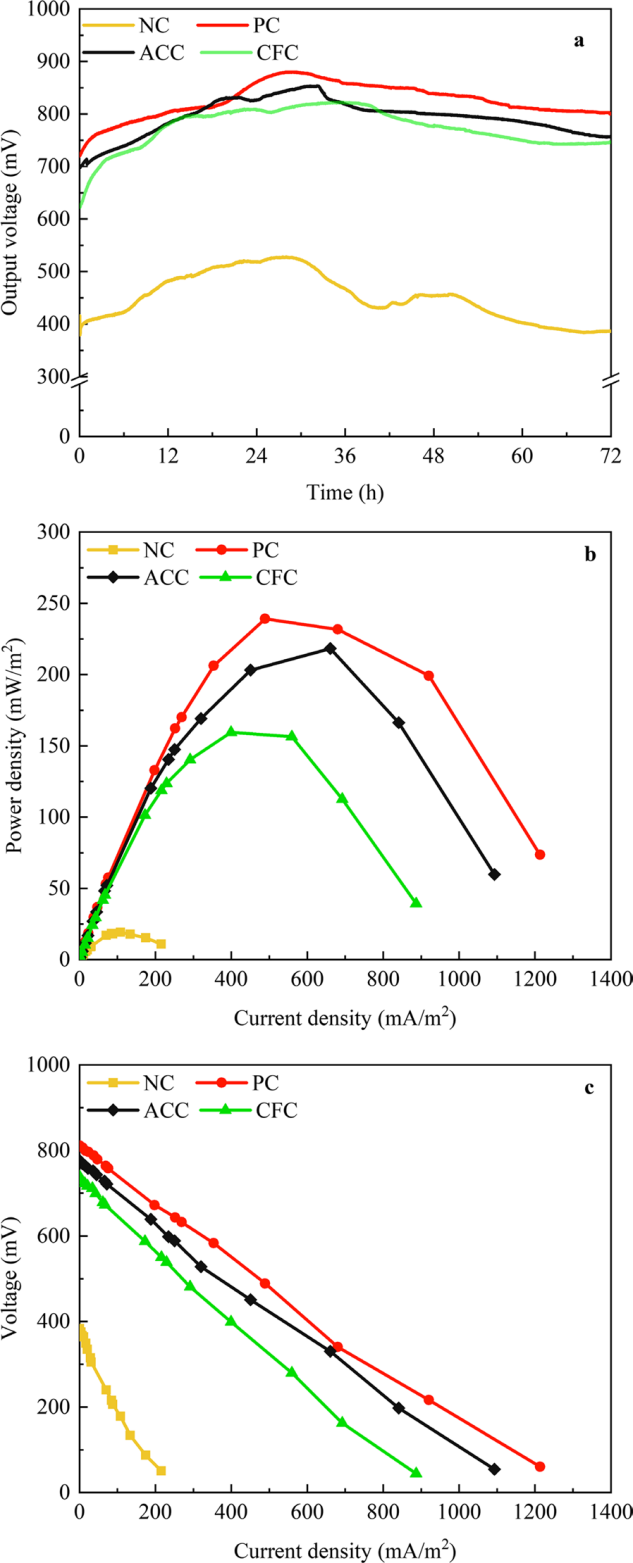
MFC electrical performance in no carrier fermentation (NC), polyurethane and carbon felt (PC), absorbent cotton and carbon felt (ACC) and cotton fiber and carbon felt (CFC) carrier solid-state fermentation. **a** output voltage curves, **b** power density curves, and **c** polarization curves

Figure 1a shows the output voltage of MFCs by NC, PC, ACC and cotton fiber and carbon felt (CFC) carrier solid-state fermentation. The output voltage of different fermentation processes had the same trend: updown–updown. *Clostridium acetobutylicum* was in the adjustment period during the first 6 h after inoculation. It synthesized a large number of substances to adapt to the new environment. Therefore, the output voltage raised rapidly. For example, the output voltage in CFC increased from 623 to 726 mV within 6 h. Then the output voltage reached a maximum at approximately 30 h. At this point, the output voltage in PC was 1.67 times that by NC. Finally, *C. acetobutylicum* entered the stability period and decline period. The output voltage decreased. Unlike normal bacteria, *C. acetobutylicum* had two peaks. This is because *C. acetobutylicum* has two physiological metabolic characteristics [5, 15]. The first peak responds to the acid-producing period and the second to the solvent producing period. However, the average output voltages of MFCs by ACSF were all higher than those by NC. The average output voltages by PC, ACC and CFC were 825 mV, 793 mV and 773 mV, respectively. The NC average output voltage was only 452 mV. It is proved that ACSF can improve MFC output voltage.

Figure 1b shows the power density of MFCs by NC, PC, ACC and CFC. MFCs by PC, ACC and CFC reached higher values than MFC by NC. The highest maximum power density was obtained from the MFC by PC, showing a maximum power density of 231 mW/m^2^. This power density was 91.77% higher than that of MFC by NC. The polarization curve was a common method to measure the internal resistance of MFCs. As shown in Fig. 1c, the internal resistance of MFCs by NC, PC, ACC and CFC were 326 Ω, 126 Ω, 134 Ω, 160 Ω, respectively. The internal resistance of MFC was closely related to the design of the battery. The ohmic resistance accounted for 83% of the total resistance in the two-chamber MFC. The ohmic resistance mainly comes from the barrier effect of the electrode, electrolyte and membrane on the electron conduction. The adsorption carrier in MFCs can reduce the conduction effect of electrolytes on electrons, thus reducing the internal resistance. Table 1 compares the performance of the dual chamber MFCs reported in the literature. These results indicated that the electrical performance of the MFCs by ACSF was appreciably improved by NC.

**Table 1:** Comparison of the performance of dual chamber MFC reported in the literature with the present study

Inoculum source	Substrate	Volume (L)	Time (d)	Anode	Power density (mW/m^2^)	References
*Microalga*	Effluent water from chocolate factory	1	18	Graphite	105.84	[33]
*Shewanella haliotis* and *Aeromonas hydrophilia*	Luria–Bertani culture	0.2	0.5	Graphite	68.51	[34]
*S. oneidensis MR-1 and Rhodococcus* sp	o-xylene	0.06	8	Carbon brush	92.5	[35]
Bacterial community within ceramic-based MFC fed with human urine	Human urine	0.06	20	Carbon veil	36.66	[36]
Anaerobic sludge	Synthetic wastewater	0.03	1.6	Graphite felt	264.5	[37]
*C. Acetobutylicum*	Synthetic medium	0.02	2	Graphite	0.33	[9]
*C. Acetobutylicum*	Artificial wastewater	0.5	9	Carbon paper	3.36	[10]
*C. acetobutylicum*	Synthetic medium	0.2	2	Carbon felt	231	This study

### Fermentation performance of MFCs in adsorption carrier solid-state fermentation

**Fig. 2:**
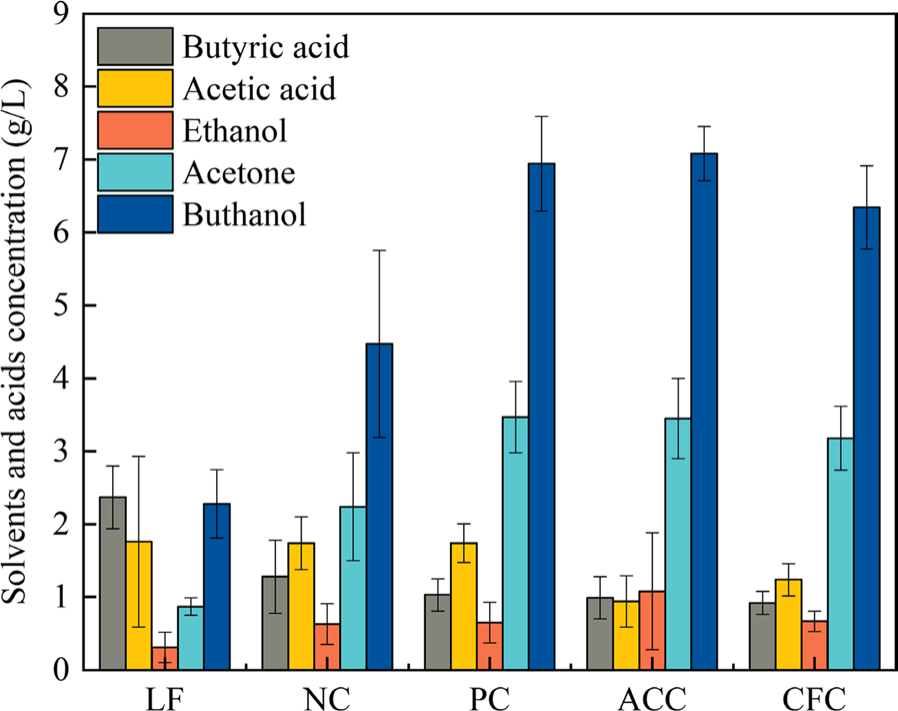
Solvents and acids concentration of liquid fermentation (LF), no carrier fermentation (NC), polyurethane and carbon felt (PC), absorbent cotton and carbon felt (ACC) and cotton fiber and carbon felt (CFC) carrier solid-state fermentation in MFC

The effect of the open circuit, closed circuit and adsorption carrier on fermentation performance was studied. The product concentration is shown in Fig. 2. The biobutanol yield in the closed circuit was 46.60% higher than that in the open circuit. The electric current can promote bacterial growth, ATP synthesis and protein expression [16]. Furthermore, the fermentation performance by NC and ACSF was compared. The solvent and biobutanol yield by ACSF was significantly higher than that by NC, and the acid yield was lower than that by NC. The solvent and biobutanol yield of ACC were 11.61 g/L and 7.08 g/L, respectively, which increased by 36.77% and 36.86% compared with NC. The carriers increase the specific surface area, provide more attachment points, and then improve the yield of butanol, which is consistent with the literature report [17]. In addition, the reason may be that the reabsorption capacity of organic acids was enhanced and the harmful substances around *C. acetobutylicum* were reduced with adsorption carriers. In addition, *C. acetobutylicum* can interact with the adsorption carrier to form a cross-linked structure to promote the formation of the cell membrane, improving the tolerance of the strain [18]. These results showed that the closed circuit and adsorption carrier had a positive effect on fermentation performance.

### Metabolic flux analysis of *C. acetobutylicum* in adsorption carrier solid-state fermentation

**Fig. 3:**
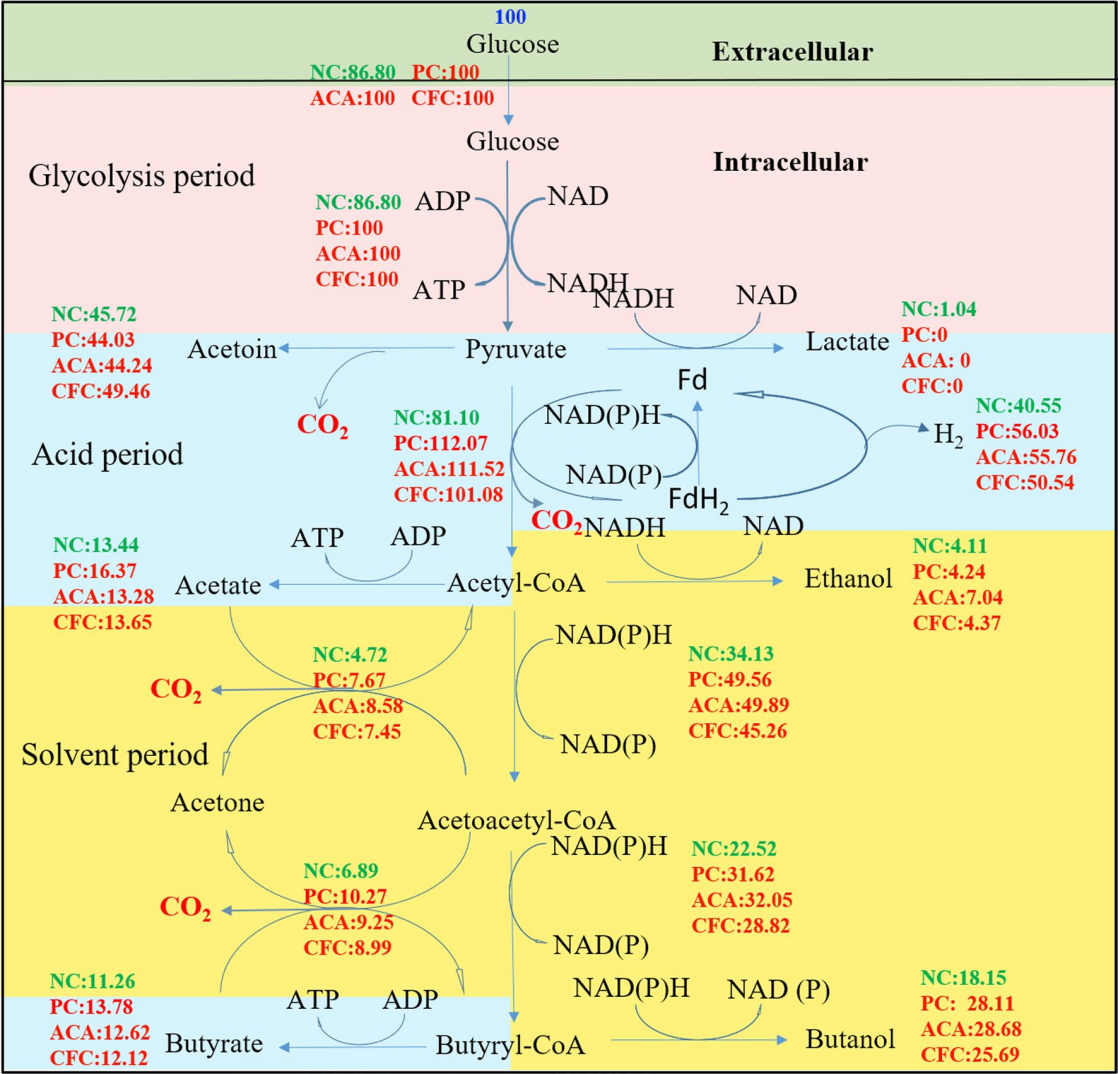
Distribution of metabolism flux by no carrier fermentation (NC), polyurethane and carbon felt (PC), absorbent cotton and carbon felt (ACC) and cotton fiber and carbon felt (CFC) carrier solid-state fermentation. Green: NC, red: ACSF

The metabolic activity of microorganisms determined the flow of electrons and protons, which affected the performance of electrical MFCs [19]. Figure 3 shows the metabolic flux distribution of *C. acetobutylicum* in different fermentation methods. As can be seen, the intracellular absorption and utilization rate of glucose was faster by ACSF. This indicated that the metabolic activity of *C. acetobutylicum* was faster by ACSF than that by NC. Then, glucose generates pyruvate through glycolysis, which is further degraded to acetyl coenzyme A and hydrogen. The hydrogen produced by ACSF is more than that by NC. The more release of hydrogen also indicates that more electrons are generated in the cell [19]. The part of the electricity in MFC was stored in acids and alcohols and other parts were used to form electric current. Electrons were mainly generated by the oxidation of glucose to carbon dioxide in anaerobic fermentation. Specifically, 1 mol CO_2_ was accompanied by 4 mol electrons. The electron yield was calculated by metabolic flux distribution. The electron production was mainly concentrated in the reaction of acetyl coenzyme A, acetone and acetoin. The electron yields by PC, ACC and CFC were 218.07, 217.83 and 210.44 mmol, respectively. The electron yield by PC was 15.54% higher than that by NC. More electrons released improved NADH yield, which further improved biobutanol yield [20]. The ACSF flux maps were obviously different from NC. Most notably, electrons were generated by the absorption of organic acids. The rate of organic acids is faster by ACSF. For example, the rate of acetic acid absorption in ACA was 1.82 times that by NC. In addition, 100% pyruvate was used to generate electrons by ACSF, while 0.52% pyruvate was used to produce lactate without electrons. These results indicated that more electrons were generated and more carbon was used for biobutanol production by ACSF.

### Cyclic voltammetry analysis of MFCs in adsorption carrier solid-state fermentation

**Fig. 4:**
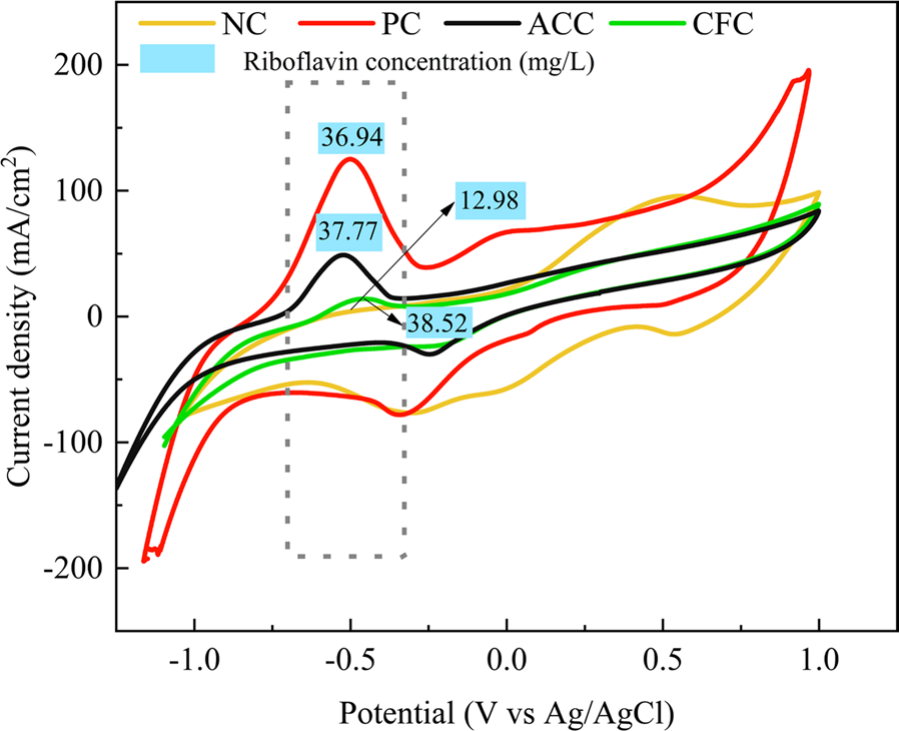
Cyclic voltammetry curves of MFCs and riboflavin concentration by no carrier fermentation (NC), polyurethane and carbon felt (PC), absorbent cotton and carbon felt (ACC) and cotton fiber and carbon felt (CFC) carrier solid-state fermentation

The CV was performed to investigate the EET between bacteria and anode in the MFC, as shown in Fig. 4. Three oxidation peaks of − 0.501 V, 0.922 V, − 0.006 V and two reduction peaks of 0.492 V, − 0.321 V were observed on the CV of PC. However, there was one oxidation peak and two reduction peaks. Since the redox peak number was proportional to the electron shuttles type [21], the CV results suggested that PC had more kinds of electron shuttles compared to the NC. It is greatly favorable to improve EET performance. The most obvious difference between ACSF and NC was that ACSF had a pair of obvious redox peaks at approximately − 0.5 V, but NC did not. The larger the peak current density of the positive response is, the better the electrical performance of the MCF [22]. The peak current densities of PC, ACC and CFC were 125.08 mA, 48.86 mA and 14.03 mA, respectively, the trend of which was consistent with the output voltage. This indicated that the EET mediated by the electron shuttle played an important role at approximately − 0.5 V. It has reported that − 0.5 V is a typical redox peak of riboflavin [23]. In addition, it has been reported that *C. acetobutyricum* can enhance the EET rate by increasing the secretion of riboflavin [24]. This indicated that the presence of free riboflavin by ACSF and ACSF can promote *C. acetobutylicum* to secrete riboflavin [23]. Riboflavin can promote EET in different ways according to different electron acceptors [25]. On the one hand, riboflavin can act as an electron shuttle to directly mediate electron transfer. On the other hand, riboflavin can act as a cofactor to improve the EET rate of other electron shuttles [26]. Therefore, Riboflavin concentration was detected. The riboflavin concentration of ACSF was almost three times that of NC. Surprisingly, the riboflavin concentrations of PC, ACC and CFC were almost the same and inversely proportional to the peak current density. This is likely because the concentration of riboflavin detected included not only extracellular free riboflavin, but also intracellular riboflavin. In addition, riboflavin can regulate the thickness of the cell membrane and exit in the cell membrane. [27]. For gram-positive bacteria, the thickening of cell membrane will hinder electron transfer [28]. In addition, a special riboflavin electron transport mechanism may be in *C. acetobutyricum* [24]. This needs further study in the future.

### Anode morphology of MFCs in adsorption carrier solid-state fermentation

**Fig. 5:**
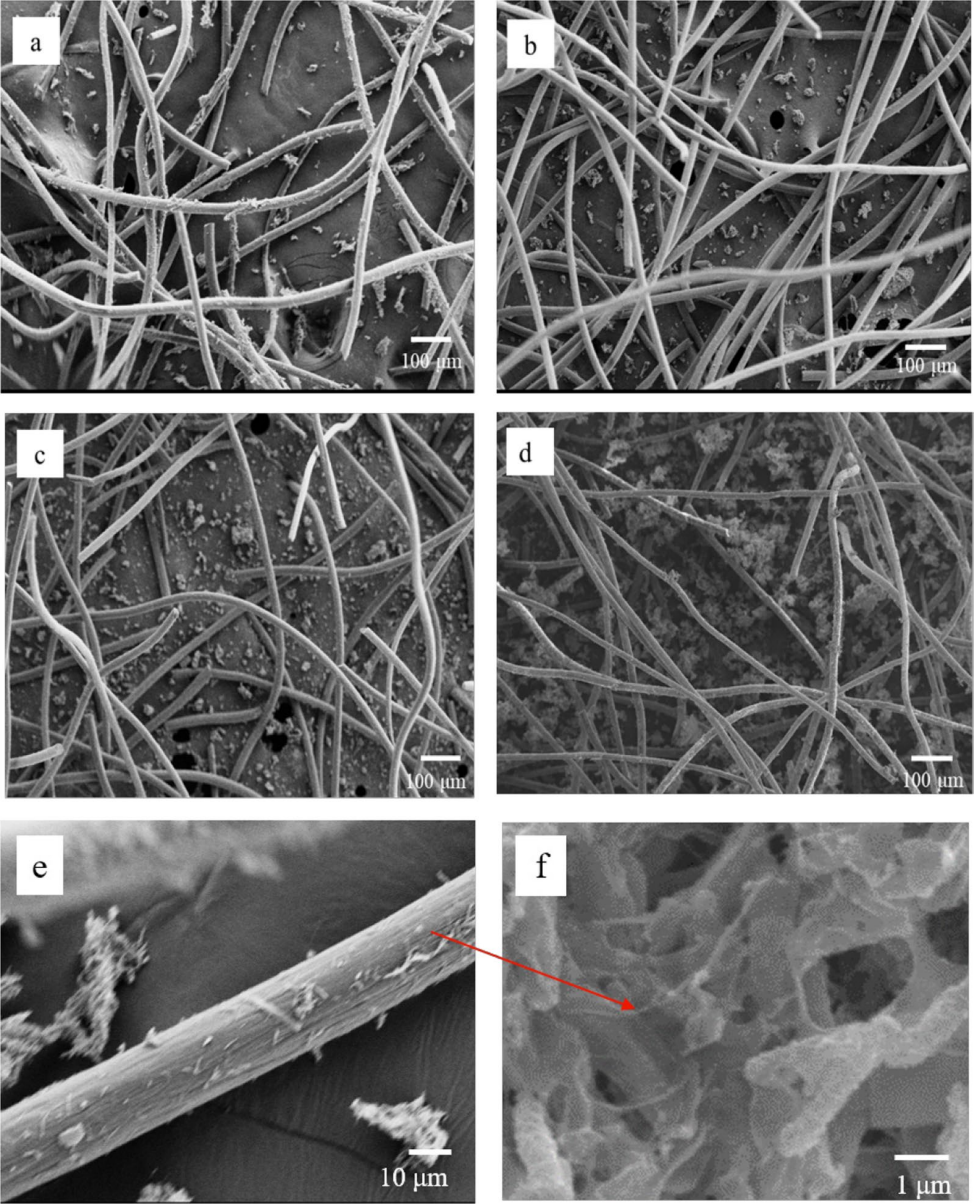
SEM image of carbon felt anode by no carrier fermentation (NC), polyurethane and carbon felt (PC), absorbent cotton and carbon felt (ACC) and cotton fiber and carbon felt (CFC) carrier solid-state fermentation. **a**, **e**, **f** NC, **b** CFC, **c** ACC, **d** PC

**Fig. 6:**
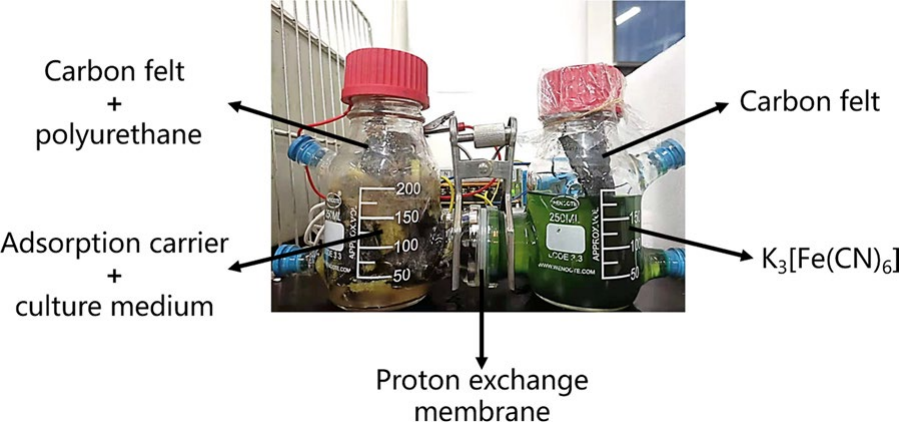
Device photograph of coupling MFC with adsorption carrier solid-state fermentation

The microstructure of the anode carbon felt is shown in Fig. 5. The *C. acetobutylicum* and metabolites were distributed in fiber surface and space between fibers after fermentation. The number of bacteria by ACSF was more than that by NC. Especially on the anode of PC, the number of bacteria was not only the largest, but also the aggregation degree was highest. Thus, the distance of electron transfer was shortened and EET efficiency was improved. The single fiber was enlarged to study the adsorption of bacteria on fiber. It can be seen from Fig. 6e that the bacteria were closely linked with carbon fiber, and the electrons were produced and transferred directly to the anode. Some bacteria gathered together to form colony and adsorbed on the electrode of PC. The distance between cells was shortened and increased signal transfer. Furthermore, the colony on the fiber was enlarged to study the relationship between the bacteria. It can be observed that the cells were fusiform. In addition, there are filamentous substances between them, which were nanowires. It twined around the outer surface of the cell and connected with other cells. Therefore, it can transfer the electron between the cells and between cell and medium [29]. It also indirectly proved that *C. acetobutylicum* can generate nanowires and transfer the electrons by it.

## Conclusions

A coupling system of biobutanol production and MFC has been built in this study. Then, the MFC power generation performance and fermentation performance were evaluated by ACSF. The power density by ACSF was 12 times higher than that by NC. In addition, the biobutanol concentration was increased from 4.47 to 7.08 g/L by ACSF. Furthermore, the mechanism of ACSF intensification method was explored. The metabolic flux analysis revealed that more electrons were generated and more carbon sources flowed to biobutanol production by ACSF. The response current of ACSF was significantly enhanced at − 0.5 V due to the high riboflavin concentration. The SEM revealed that nanowires existed and that the bacteria density and aggregation degree were improved by ACSF.

## Materials and methods

### Microorganism and media

*Clostridium acetobutylicum* was purchased from China General Microbiological Culture Collection Center. It was cultured in 6% corn liquid at 37 °C for 20 h and then inoculated in MFCs. The medium contained 60 g/L C_6_H_12_O_6_, 3.68 g/L (NH_4_)_2_SO_4_, 1.768 g/L KH_2_PO_4_, 2.938 g/L K_2_HPO_4_, 2 g/L Ca(OH)_2_, 6 g/L yeast extract, 3 g/L tryptone and 10 mL/L microelements solution (2.4 g/LNa_2_MoO_4_, 1.5 g/LCaCl_2_, 27 g/LFeCl_3_, 1 g/L CuSO_4_, 0.29 g/L ZnSO_4_, 1.7 g/L MnSO_4_·H_2_0, 12 g/L MgSO_4_·7H_2_O, 1 g/L p-aminobenzoic acid and 1 g/L biotin).

### The preparation of adsorption carrier and electrode

The carbon felt was immersed in 1 mol/L hydrochloric acid for 24 h and then washed with water to neutral (pH = 7), followed by drying. The carbon felt, polyurethane, absorbent cotton and cotton fiber were cut into two sizes of 40 × 100 × 3 mm and 20 × 20 × 3 mm. The sizes of carrier and electrode were 20 × 20 × 3 mm and 40 × 100 × 3 mm, respectively. ACC was the combination of carbon felt and absorbent cotton. PC was the combination of carbon felt and polyurethane. CFC was the combination of carbon felt and cotton fiber.

### The construction of MFC

The microbial fuel cell reactors with two chambers were purchased from Shanghai Wenshen Experimental Equipment Company. The cathode chamber was 200 mL of 25 mM K_3_[Fe(CN)_6_] and the anode chamber consisted of 40 adsorption carriers, carbon felt electrode, 200 mL culture medium and 20 mL seed liquid. The electrode was located in the middle of the anode chamber, and the adsorption carriers fill the whole anode chamber. The culture medium liquid was bounded with adsorption carrier. The anode chamber and cathode chamber were separated by DuPont proton exchange membrane N-17. The electrode is connected with 10 KΩ through crocodile clamp wire. The photograph of MFC is shown in Fig. 6.

### The electrochemical performance measurement of MFC

The voltage was collected by a data acquisition (China Shaanxi Ruikong Intelligent Technology Co., Ltd.) and recorded every 1 min. The power density and polarization curve were obtained by changing the external resistance (10 Ω–300 KΩ) at 48 h. An electrochemical workstation (Wuhan Kesite Electrochemical Workstation) was used to measure the CV. The CV measurement was carried out in a three-electrode mode with the working electrode, the reference electrode Ag/AgCl, the counter electrode Pt. The scanning range is − 1.0 V to + 1.0 V and the scanning speed is 50 mV/s. All experiments were carried out three times and the average values were taken.

### The production measurement and metabolic flux analysis

Glucose and lactic acid concentration were determined by high performance liquid chromatography (Agilent 1200, USA). The chromatographic conditions were as described previously [30]. The detection conditions of lactic acid and glucose are different only in the detector temperature and column temperature. The detector temperature of lactic acid was 50℃, the column temperature was 65 °C.

Acetic acid, butyric acid, acetone, ethanol and biobutanol were determined by gas chromatography (Agilent 7980A, USA) with innowax 19091 N-113, 30 m × 0.32 mm × 25 mm, hydrogen flame detector. The chromatographic conditions: the total pressure of nitrogen, hydrogen and air, 0.12 MPa, flow rate ratio of nitrogen and hydrogen, 10:1, injector temperature, 250 °C; detector temperature, 250 °C, the temperature program: 85 °C for 4.5 min, from 85 to 170 °C at a rate of 20 °C/min, 170 °C for 2.5 min. The isobiobutanol was used as the internal standard and the volume ratio of internal standard to sample was 1:1.

The riboflavin concentration was obtained by measuring optical density at 444 nm. The treatment and calculation method were as described previously [31]. All experiments were carried out three times and the average values were taken.

The metabolic pathway and stoichiometry were determined according to [32]. The metabolic network and model were constructed. It was based on the pseudo steady state assumption that the concentration of intermediate metabolite was 0. The formula is as follows.

1 $$S \times v = c$$

where *S* is the matrix of *m* × *n*, *m* is the number of metabolites, *n* is the number of reaction equations, *v* is reaction rate and *c* is the concentration of metabolites.

### The surface morphology of anode

The middle part of the anode was immersed in 2.5% glutaraldehyde solution for 12 h at 4 °C. Then the anode was immersed in deionized water for 10 min. After that it was successively put in 50%, 70%, 90% and 100% ethanol for 10 min. Finally, it was immersed in 1:1 (v/v) ethanol/tert butyl alcohol for 10 min. Repeat for three times. Store it at − 20 °C for 24 h and freeze dry it for 12 h. The scanning electron microscope (SEM, Ultim MAX 300) was used to investigate anode surface morphology after spray gold treatment.

## Data Availability

All data generated or analysed during this study are included in this article.

## Abbreviations

ACC: Absorbent cotton and carbon felt carrier solid-state fermentation
ACSF: Adsorption carrier solid-state fermentation
*C. acetobutylicum*: *Clostridium* *acetobutylicum* ATCC824
CFC: Cotton fiber and carbon felt carrier solid-state fermentation
EET: Extracellular electron transport
MFC: Microbial fuel cell
NC: No carrier fermentation
PC: Polyurethane and carbon felt carrier solid-state fermentation
